# Supplementary material for: Effects of presenilin-1 familial Alzheimer’s disease mutations on γ-secretase activation for cleavage of amyloid precursor protein
Source: Commun Biol. 2023 Feb 14;6:174. doi: 10.1038/s42003-023-04539-1 (PMC9929099; doi:10.1038/s42003-023-04539-1)
Supplement: Supplementary file 2 — Supplementary Information [file 42003_2023_4539_MOESM2_ESM.pdf]

Supplementary Information

Effects of Presenilin-1 Familial Alzheimer's Disease Mutations on γ-Secretase Activation for Cleavage of Amyloid Precursor Protein

Hung N. Do[1,#], Sujan Devkota[2,#], Apurba Bhattarai[1], Michael S. Wolfe[2,*], and Yinglong Miao[1,*]

[1]Center for Computational Biology and Department of Molecular Biosciences; [2]Department of Medicinal Chemistry, School of Pharmacy, University of Kansas, Lawrence, Kansas 66047

[#] These authors contributed equally to this work

*To whom correspondence should be addressed: mswolfe@ku.edu and miao@ku.edu

Supplementary Figure 1. Time courses of distances between PS1 residues D257 (atom Cγ) and D385 (atom Cγ) **(a)**, PS1 residue D385 (protonated oxygen) and APP residue V50 (carbonyl oxygen) **(b)**, PS1 residue D385 (protonated oxygen) and APP L49 (carbonyl oxygen) **(c)**, PS1 residue D385 (protonated oxygen) and APP T48 (carbonyl oxygen) (**d**) in the WT γ-secretase.

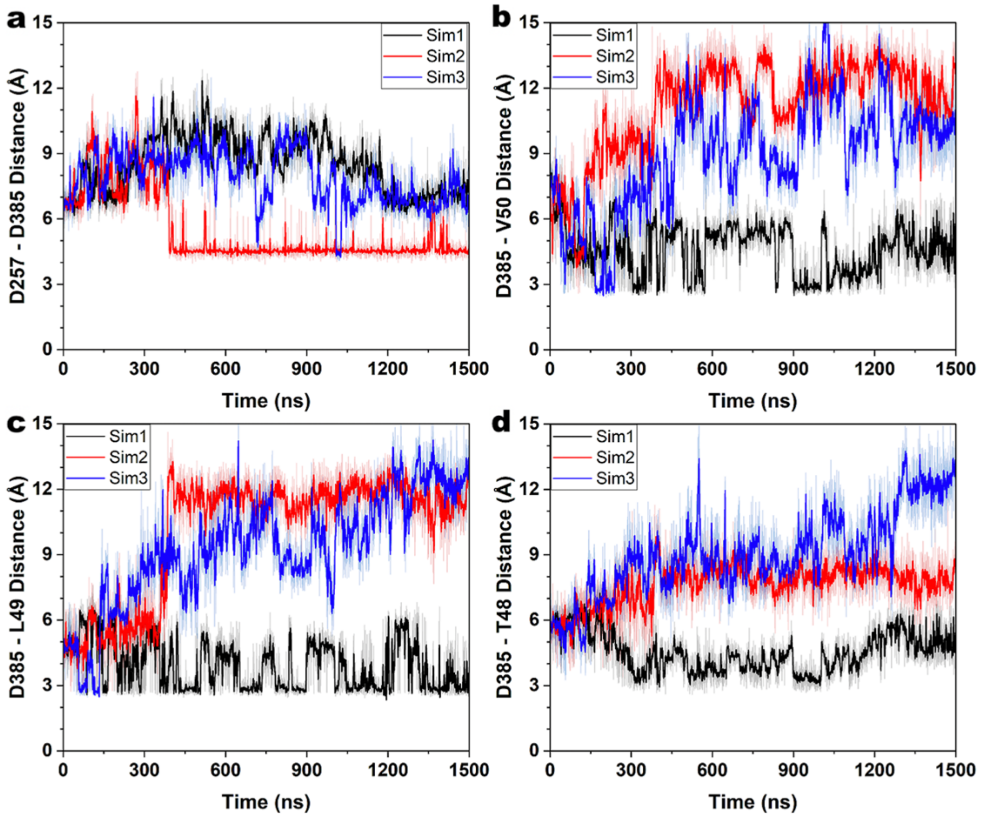

Supplementary Figure 2. Time courses of distances between PS1 residues D257 (atom Cγ) and D385 (atom Cγ) **(a** and **b)**, PS1 residue D385 (protonated oxygen) and APP residue V50 (carbonyl oxygen) **(c** and **d)**, PS1 residue D385 (protonated oxygen) and APP residue L49 (carbonyl oxygen) **(e** and **f)**, PS1 residue D385 (protonated oxygen) and APP residue T48 (carbonyl oxygen) **(g** and **h)** in the P117L **(a, c, e,** and **g)** and L286V **(b, d, f,** and **h)** PS1 FAD mutant γ-secretase.

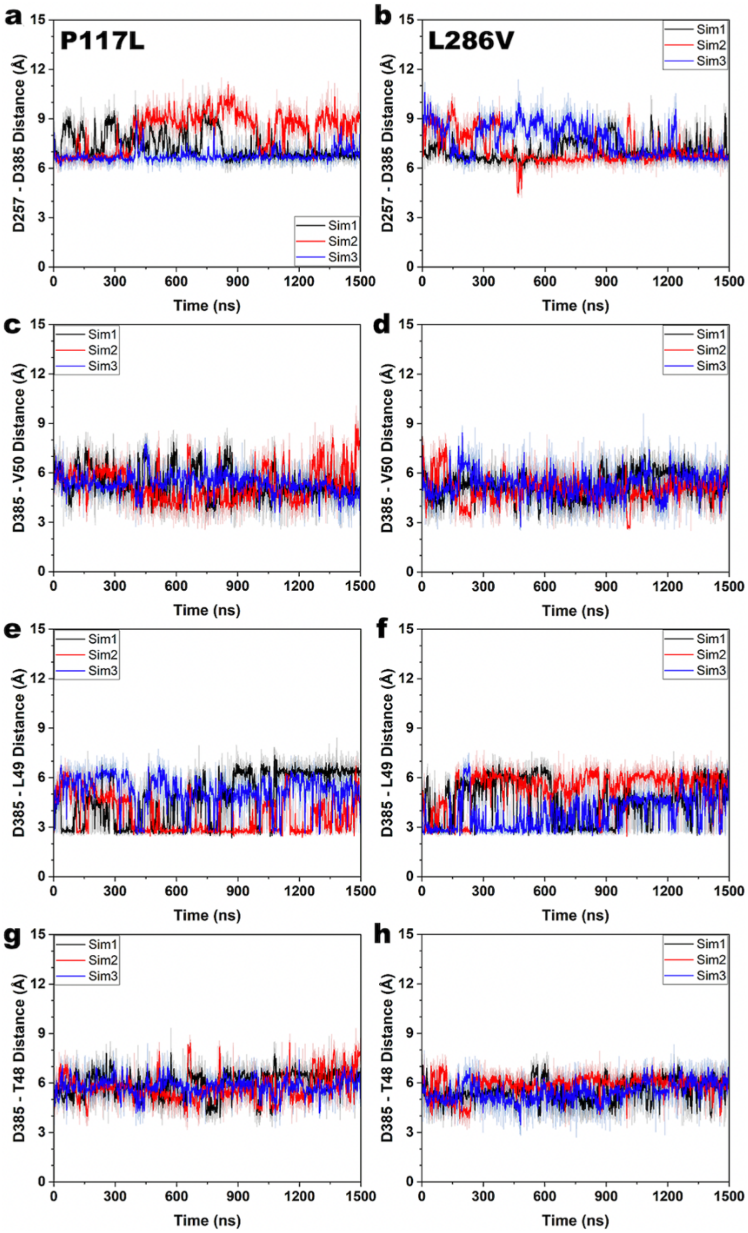

Supplementary Figure 3. Time courses of distances between PS1 residues D257 (atom Cγ) and D385 (atom Cγ) **(a** and **b)**, PS1 residue D385 (protonated oxygen) and APP residue V50 (carbonyl oxygen) **(c** and **d)**, PS1 residue D385 (protonated oxygen) and APP residue L49 (carbonyl oxygen) **(e** and **f)**, PS1 residue D385 (protonated oxygen) and APP residue T48 (carbonyl oxygen) **(g** and **h)** in the I143T **(a, c, e,** and **g)** and G384A **(b, d, f,** and **h)** PS1 FAD mutant γ-secretase.

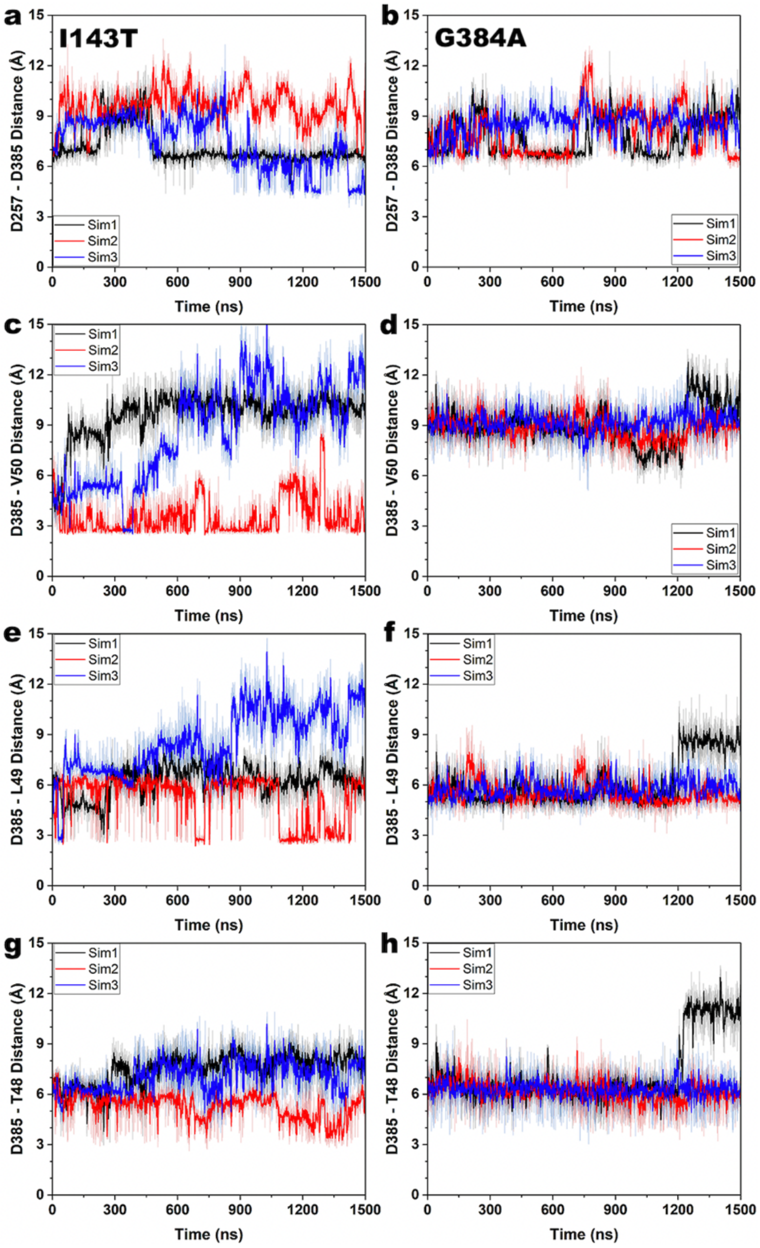

Supplementary Figure 4. Time courses of distances between PS1 residues D257 (atom Cγ) and D385 (atom Cγ) **(a** and **b)**, PS1 residue D385 (protonated oxygen) and APP residue V50 (carbonyl oxygen) **(c** and **d)**, PS1 residue D385 (protonated oxygen) and APP residue L49 (carbonyl oxygen) **(e** and **f)**, PS1 residue D385 (protonated oxygen) and APP residue T48 (carbonyl oxygen) **(g** and **h)** in the L166P **(a, c, e,** and **g)** and L435F **(b, d, f,** and **h)** PS1 FAD mutant γ-secretase.

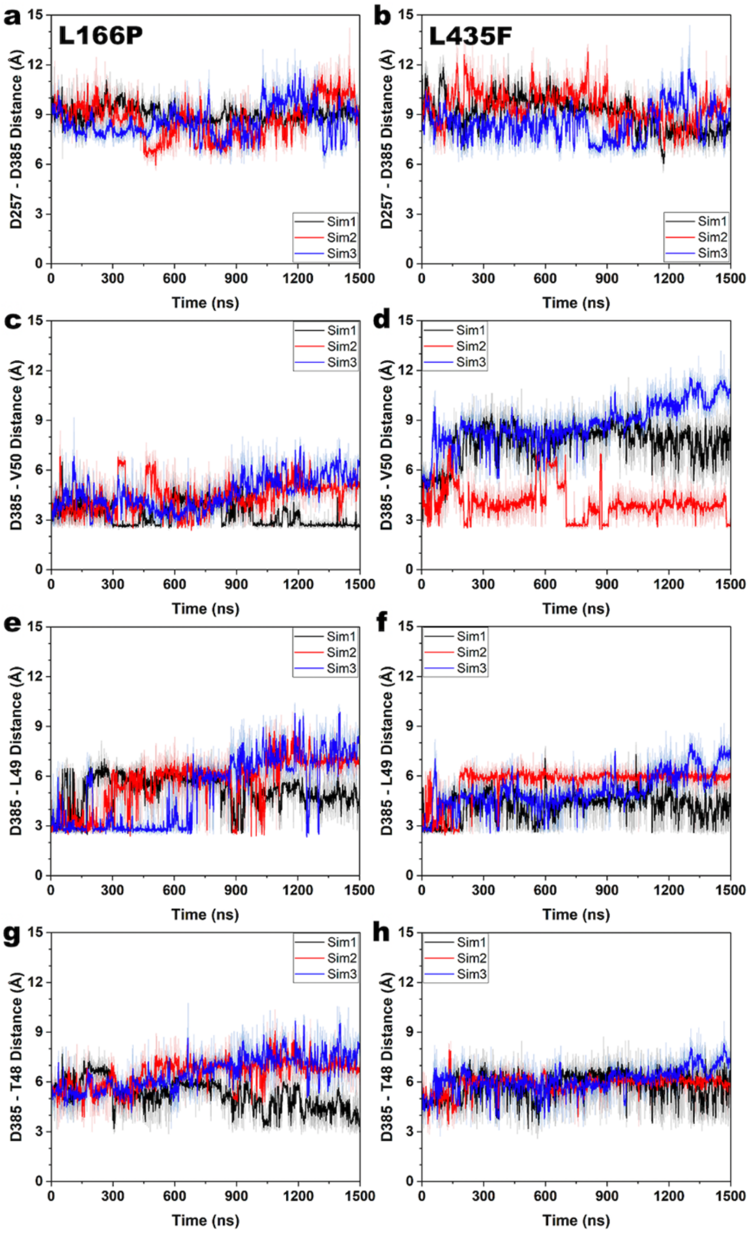

Supplementary Figure 5. Intermediate low-energy conformational states of WT and PS1 FAD mutant γ-secretase compared to "Active" WT conformation. The "Inhibited" (**a**), "I1" (**b**), "I2" (**c**), "I3" (**d**), "I4" (**e**), and "I5" (**f**) low-energy conformational state compared to "Active" WT conformational state. The transmembrane domains 1 to 9 are labeled TM1–TM9.

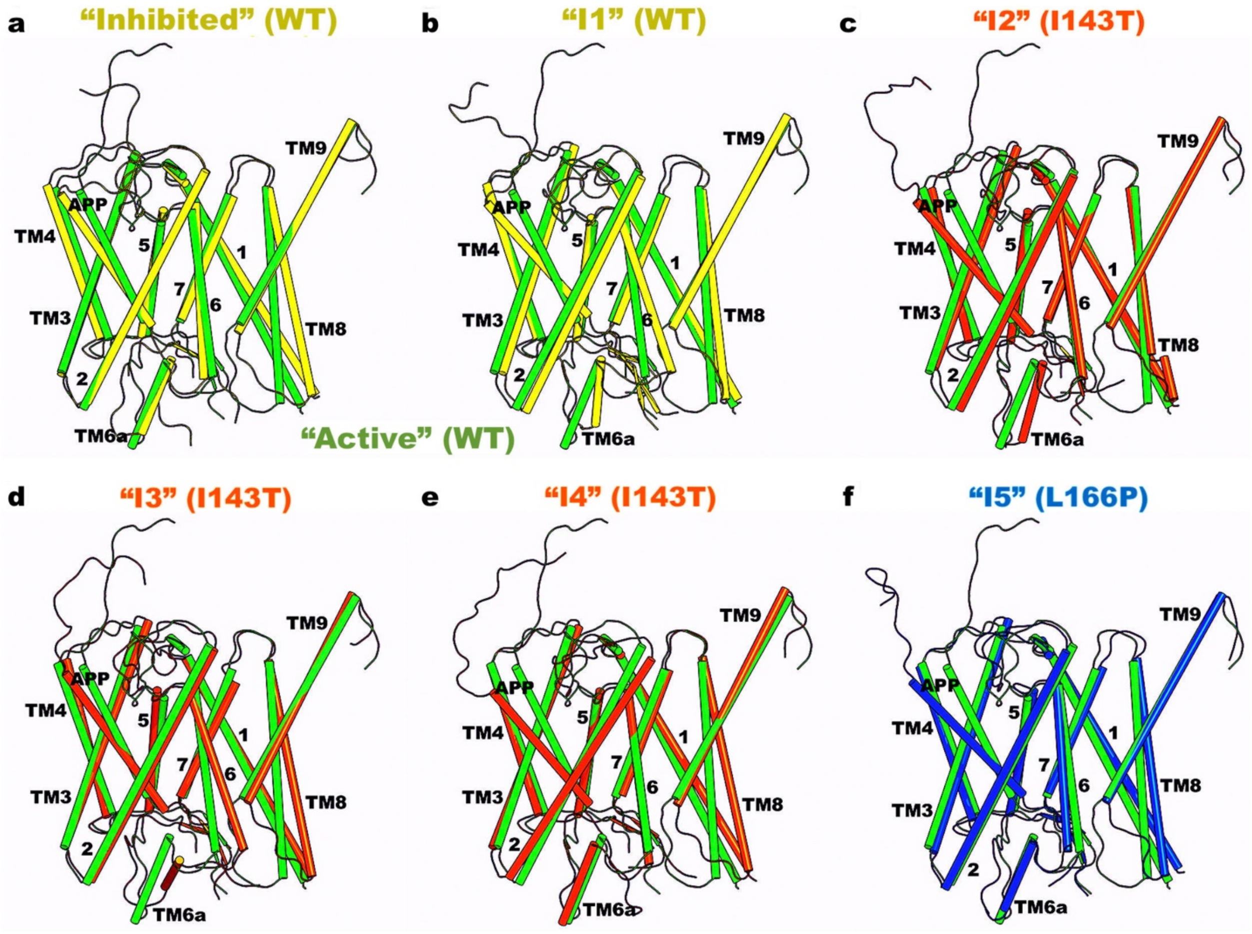

Supplementary Figure 6. Conformations of the APP substrate in the intermediate low-energy conformational states of WT and PS1 FAD mutant γ-secretase compared to "Active" WT conformation.

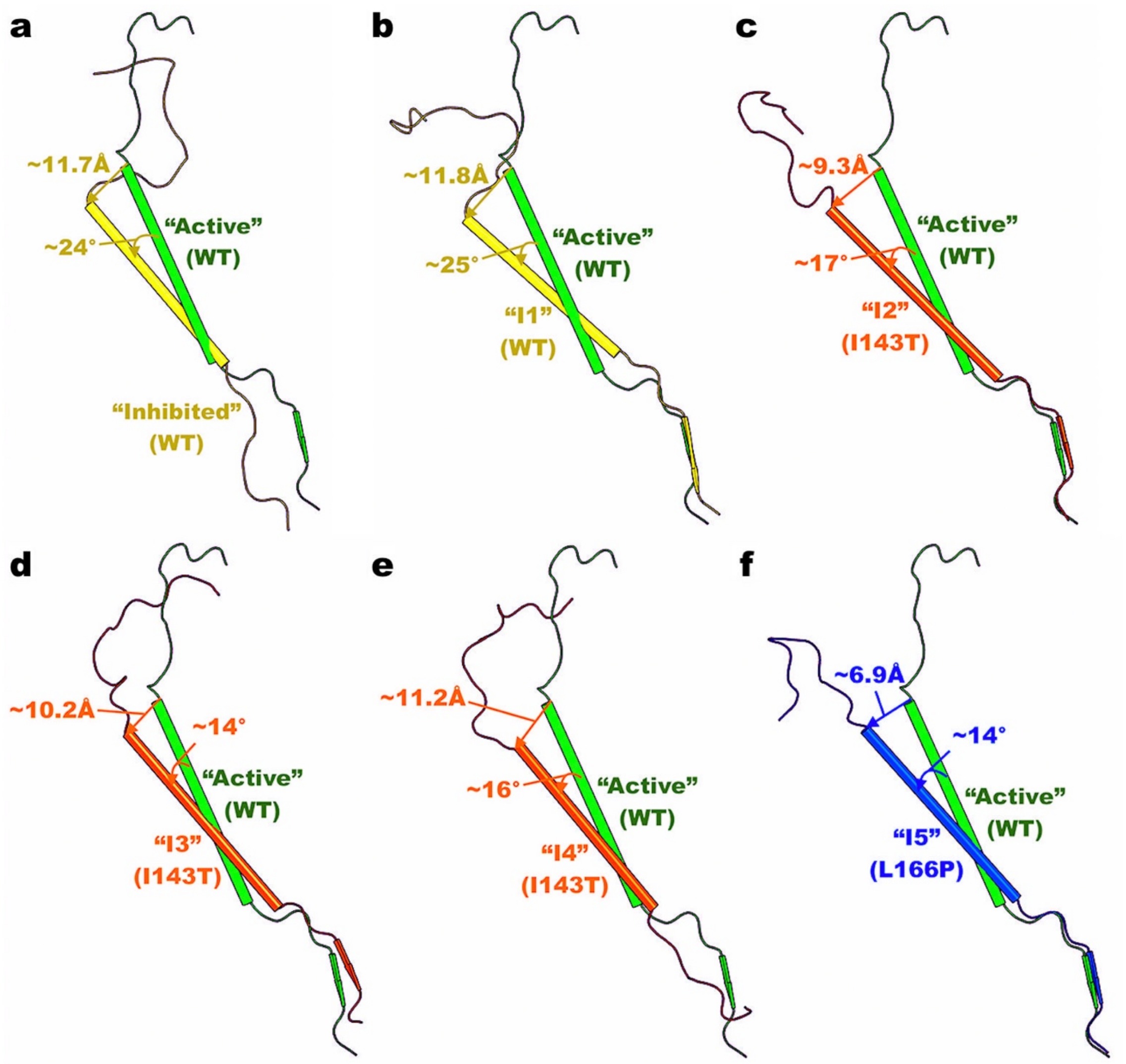

Supplementary Figure 7. Conformations of TM6a, TM7, and APP in the intermediate low-energy conformational states of WT and PS1 FAD mutant γ-secretase compared to “Active” WT conformation.

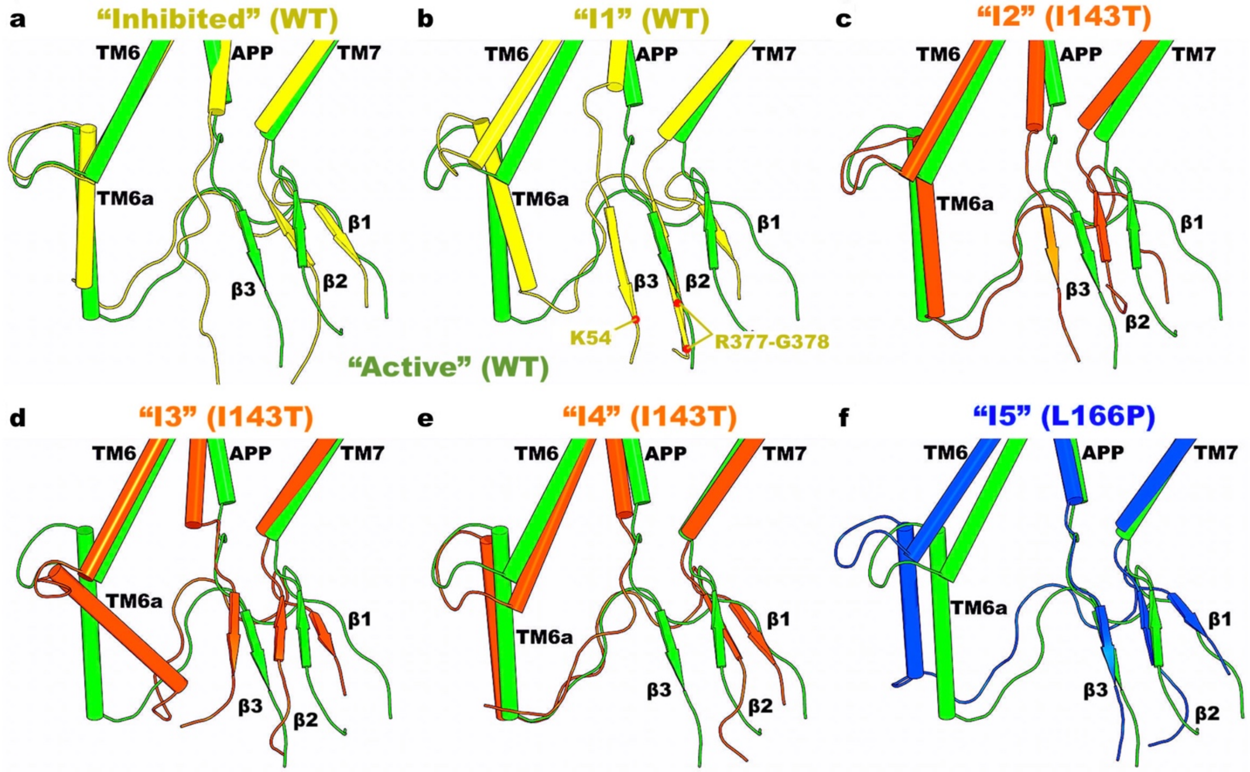

Supplementary Figure 8. Conformations of TM8 in the intermediate low-energy conformational states of WT and PS1 FAD mutant γ-secretase compared to "Active" WT conformation.

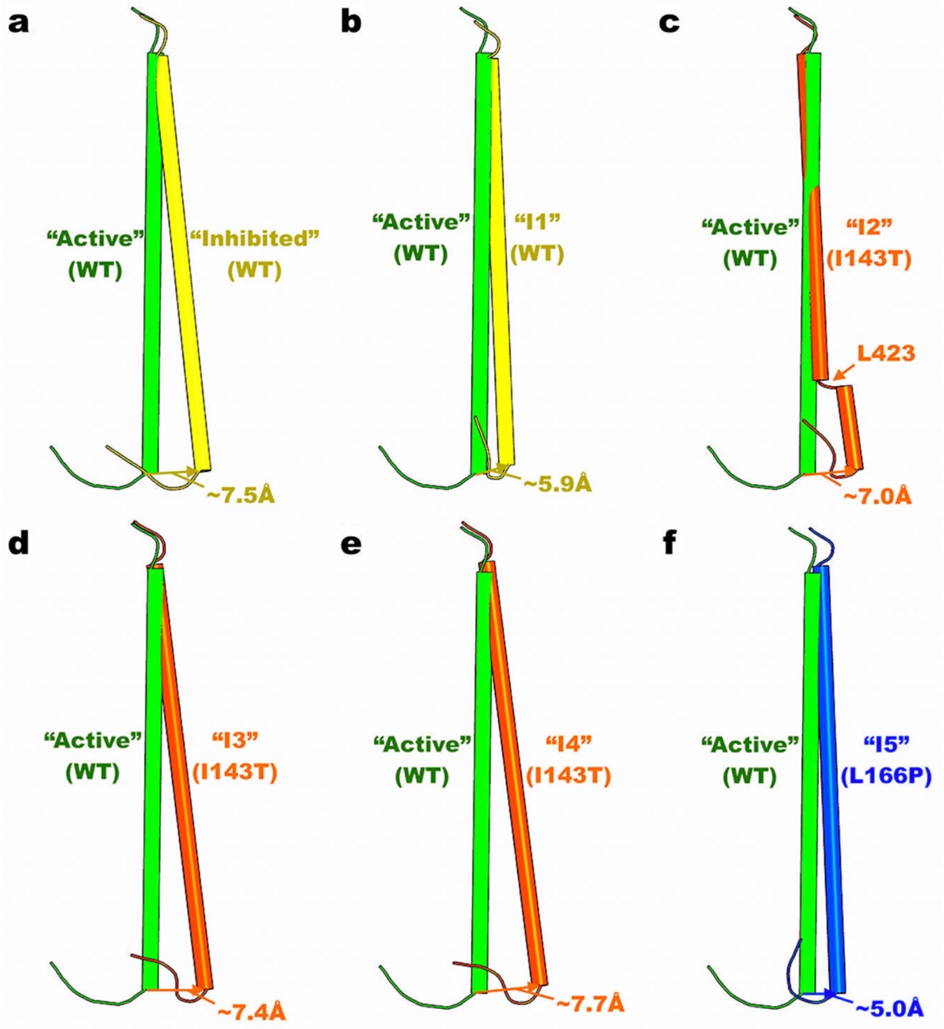

Supplementary Figure 9. Dynamics of the nucleophilic water molecules during the ε-cleavage of APP by WT γ-secretase. (a) Time courses of the distances between PS1 residues D257 (atom Cγ) and D385 (atom Cγ) (red) and PS1 residue D385 (protonated oxygen) and APP residue L49 (carbonyl oxygen). **(b)** 2D free energy profile of the distance between PS1 residues D257 (atom Cγ) and D385 (atom Cγ) and distance between PS1 residue D257 (atom OD1) and nucleophilic water (atom O). **(c)** 2D free energy profile of the distance between PS1 residue D257 (atom OD1) and nucleophilic water (atom O) and distance between PS1 residue D385 (atom OD1) and nucleophilic water (atom O). The 100ns GaMD simulation was carried out starting from the 1200ns checkpoint of Sim1 WT γ-secretase, with the coordinates of all atoms saved.

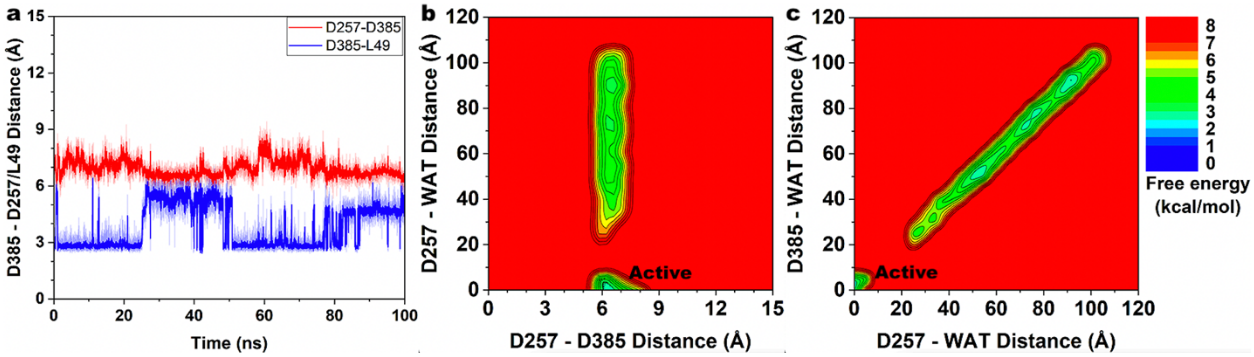

Supplementary Figure 10. Time courses of the APP secondary structures in the WT PS1 calculated from the other two independent GaMD simulations apart from the one plotted in **Figure 5**.

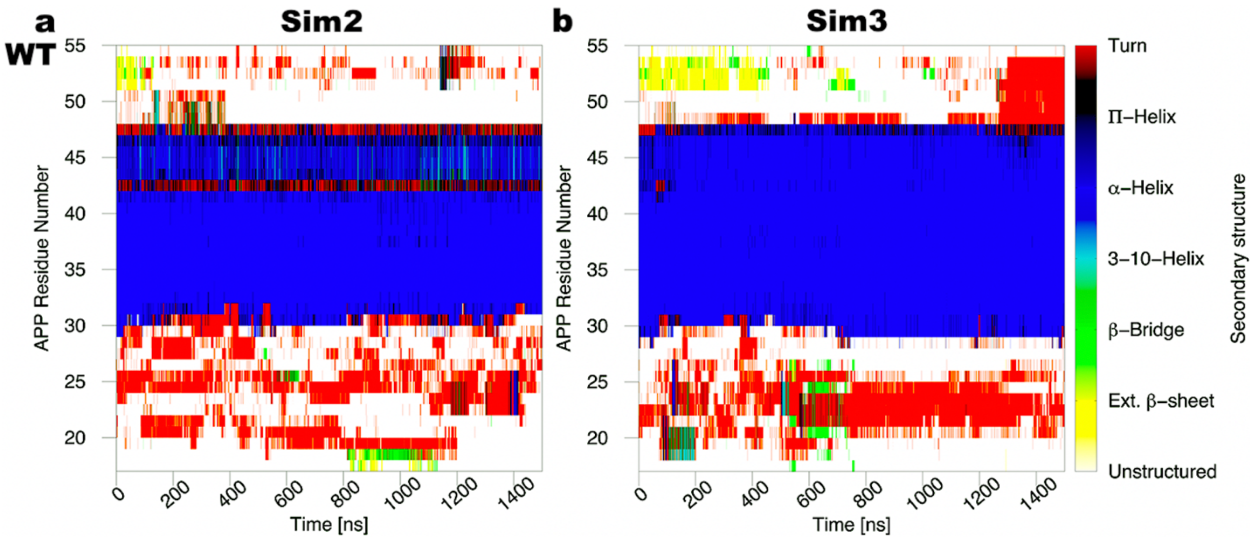

Supplementary Figure 11. Time courses of the APP secondary structures in the P117L **(a-b)** and L286V **(c-d)** PS1 calculated from the other independent GaMD simulations apart from the ones plotted in **Figure 5**.

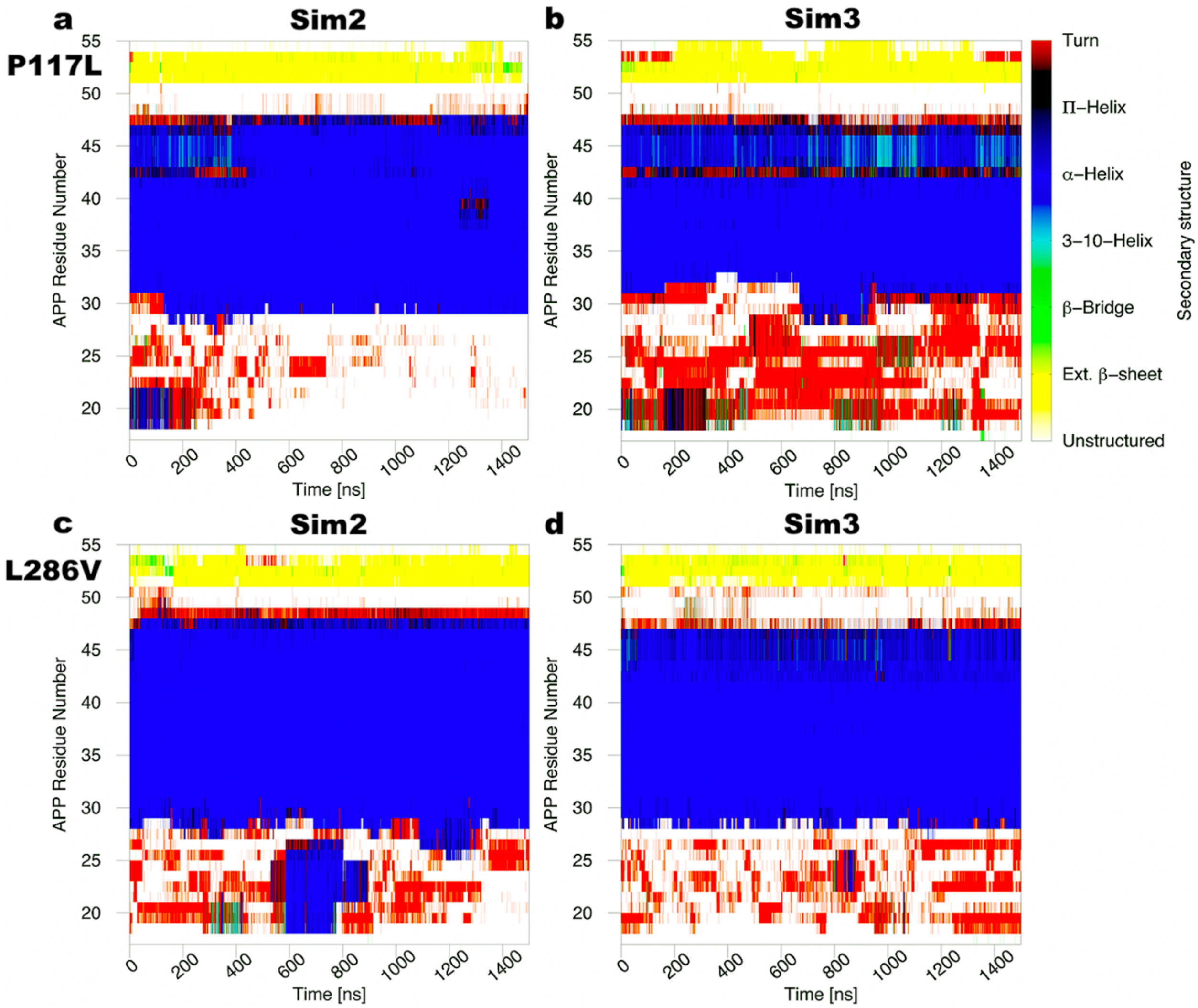

Supplementary Figure 12. Time courses of the APP secondary structures in the I143T **(a-b)** and G348A **(c-d)** PS1 calculated from the other independent GaMD simulations apart from the ones plotted in **Figure 5**.

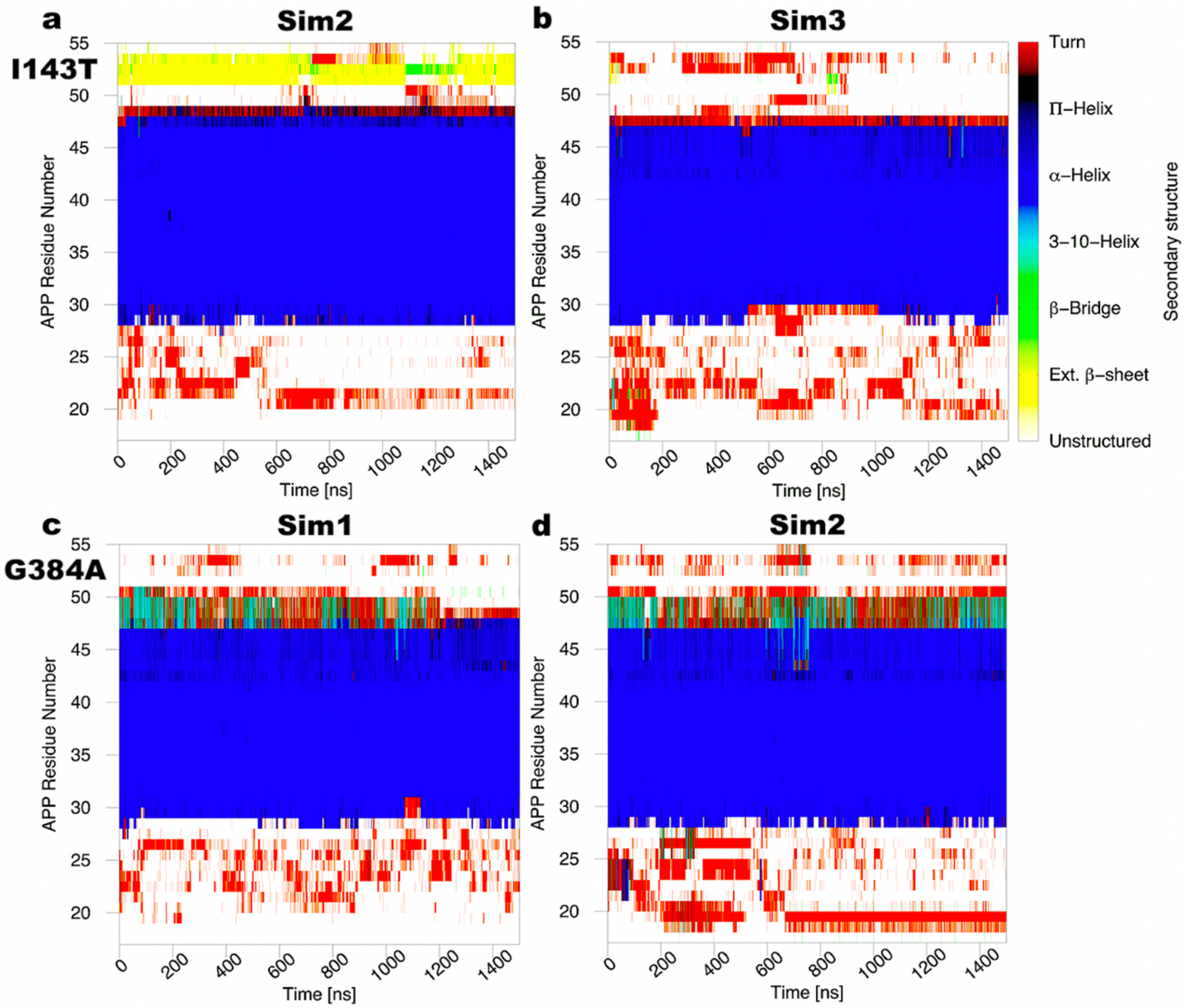

Supplementary Figure 13. Time courses of the APP secondary structures in the L166P **(a-b)** and L435F **(c-d)** PS1 calculated from the other independent GaMD simulations apart from the ones plotted in **Figure 5**.

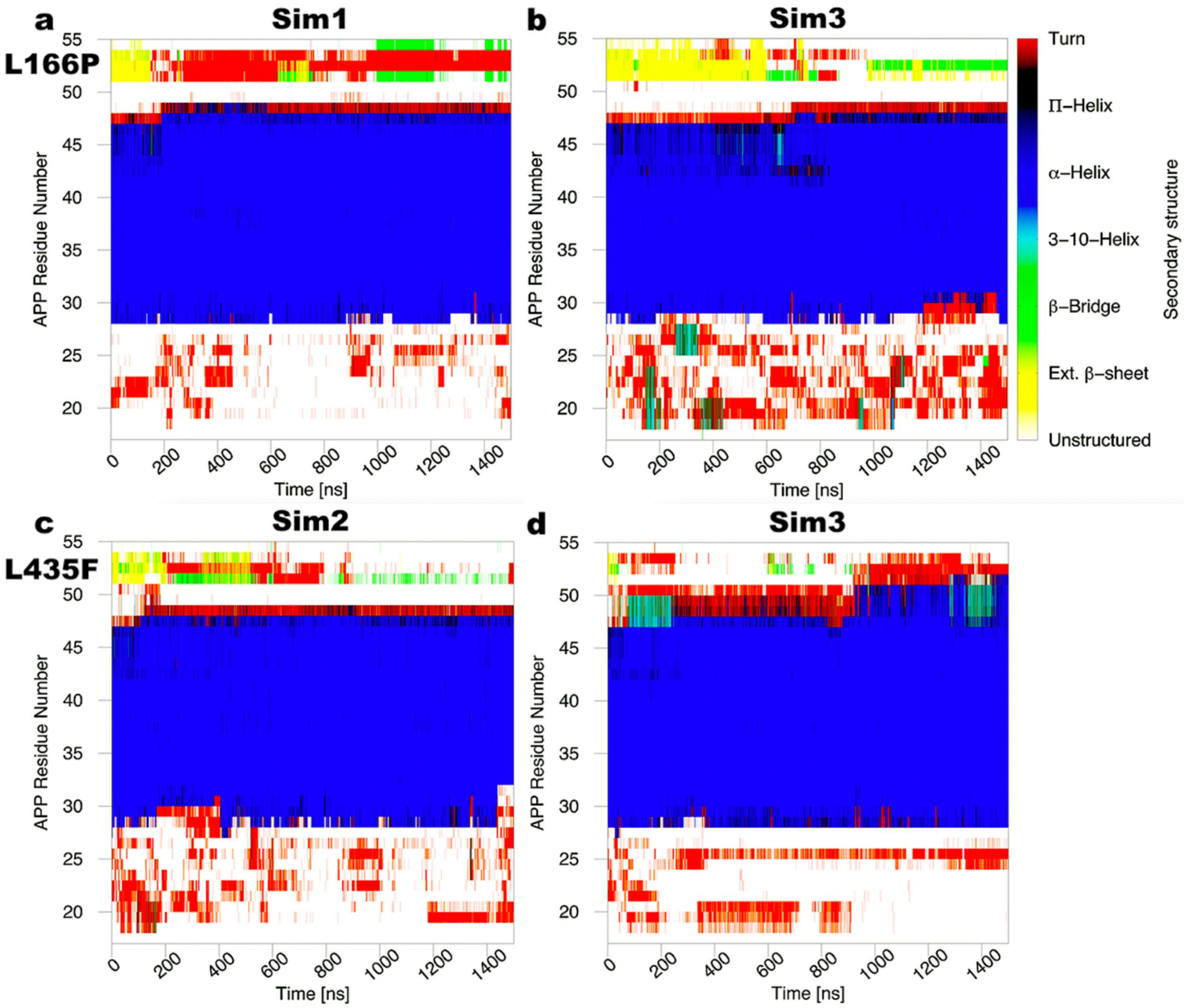

Supplementary Figure 14. Time courses of distances between PS1 residues D257 (atom Cγ) and D385 (atom Cγ) **(a)**, PS1 residue D385 (protonated oxygen) and APP residue V50 (carbonyl oxygen) **(b)**, PS1 residue D385 (protonated oxygen) and APP residue L49 (carbonyl oxygen) **(c)**, PS1 residue D385 (protonated oxygen) and APP residue T48 (carbonyl oxygen) **(d)** calculated from cMD simulations of the WT, P117L, I143T, and L166P PS1 FAD mutant γ-secretase.

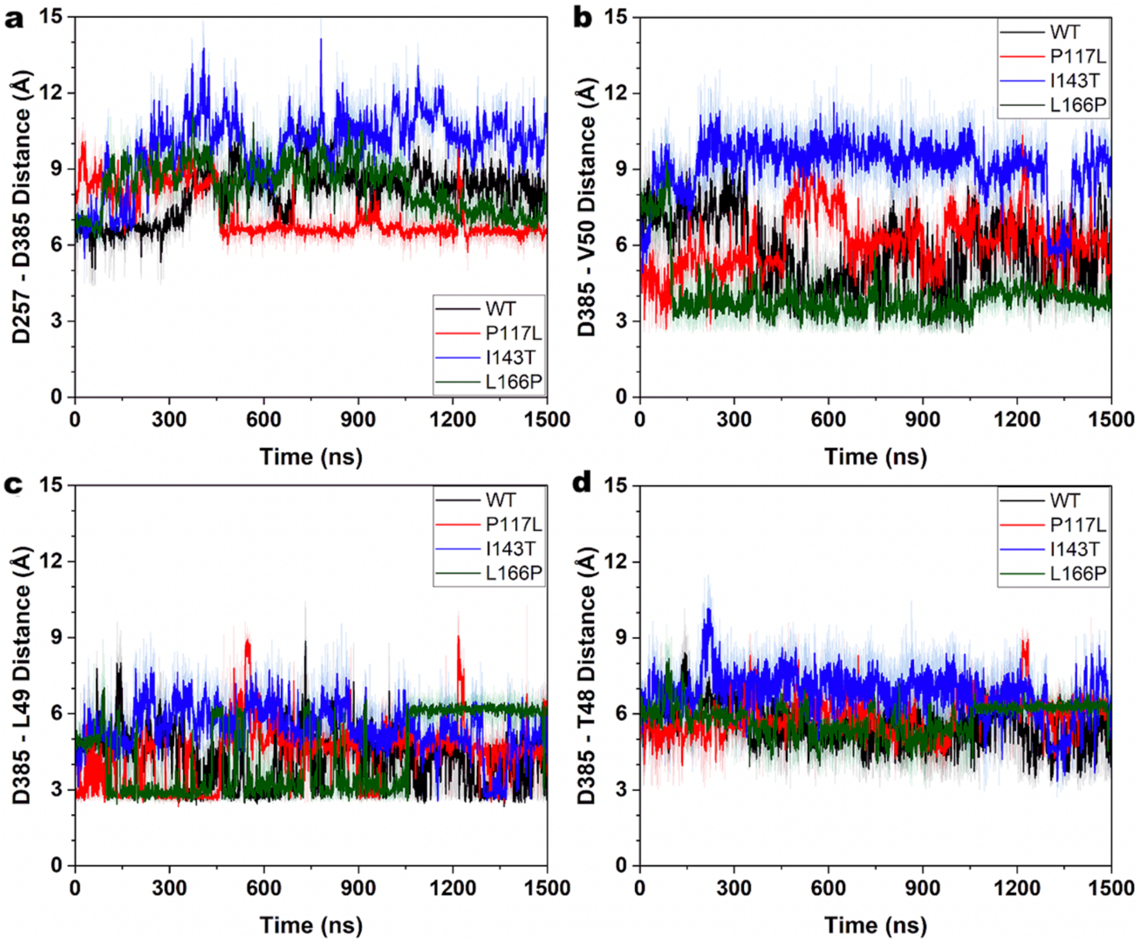

Supplementary Figure 15. 2D free energy profiles of the distance between PS1 residues D257 (atom Cγ) and D385 (atom Cγ) and distance between PS1 residue D385 (protonated oxygen) and APP residue L49 **(a-d)**, V50 **(e-h)**, or T48 **(i-l)** (carbonyl oxygen) calculated from the cMD simulations of the WT **(a, e,** and **i)**, P117L **(b, f,** and **j)**, I143T **(c, g,** and **k)**, and L166P **(d, h,** and **l)** FAD mutants of APP bound γ-secretase. The low-energy conformational states are labeled "Active" and "I2"–"I5".

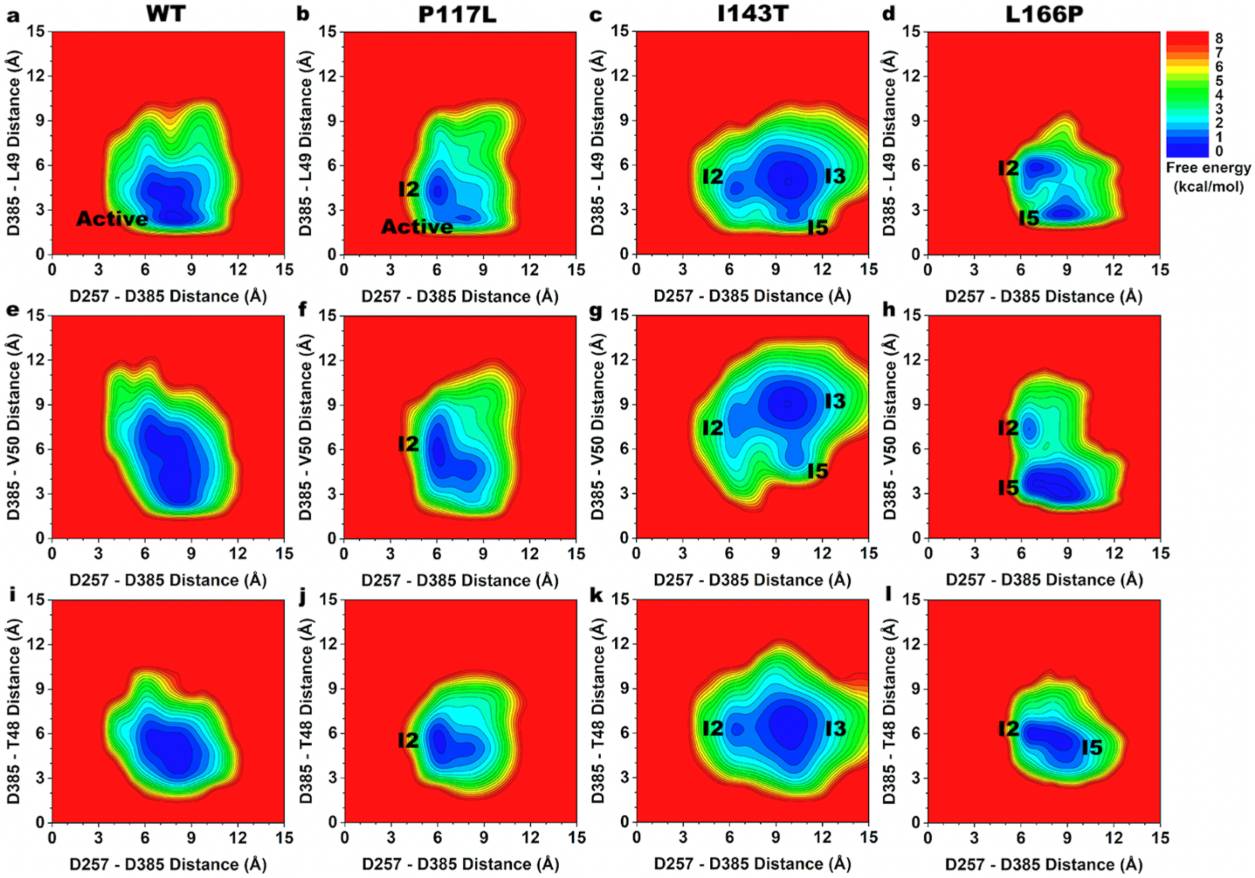

Supplementary Figure 16. 2D free energy profiles of the distance between PS1 residues D257 (atom Cγ) and D385 (atom Cγ) and distance between PS1 residue D385 (protonated oxygen) and APP residue V50 **(a-g)** or T48 **(h-n)** (carbonyl oxygen) calculated from the GaMD simulations of the WT **(a** and **h)** and P117L **(b** and **i)**, I143T **(c** and **j)**, L166P **(d** and **k)**, G384A **(e** and **l)**, L435F **(f** and **m)**, and L286V **(g** and **n)** FAD mutants of APP bound γ-secretase. The low-energy conformational states are labeled to correspond to "Inhibited" and "I1"–"I5".

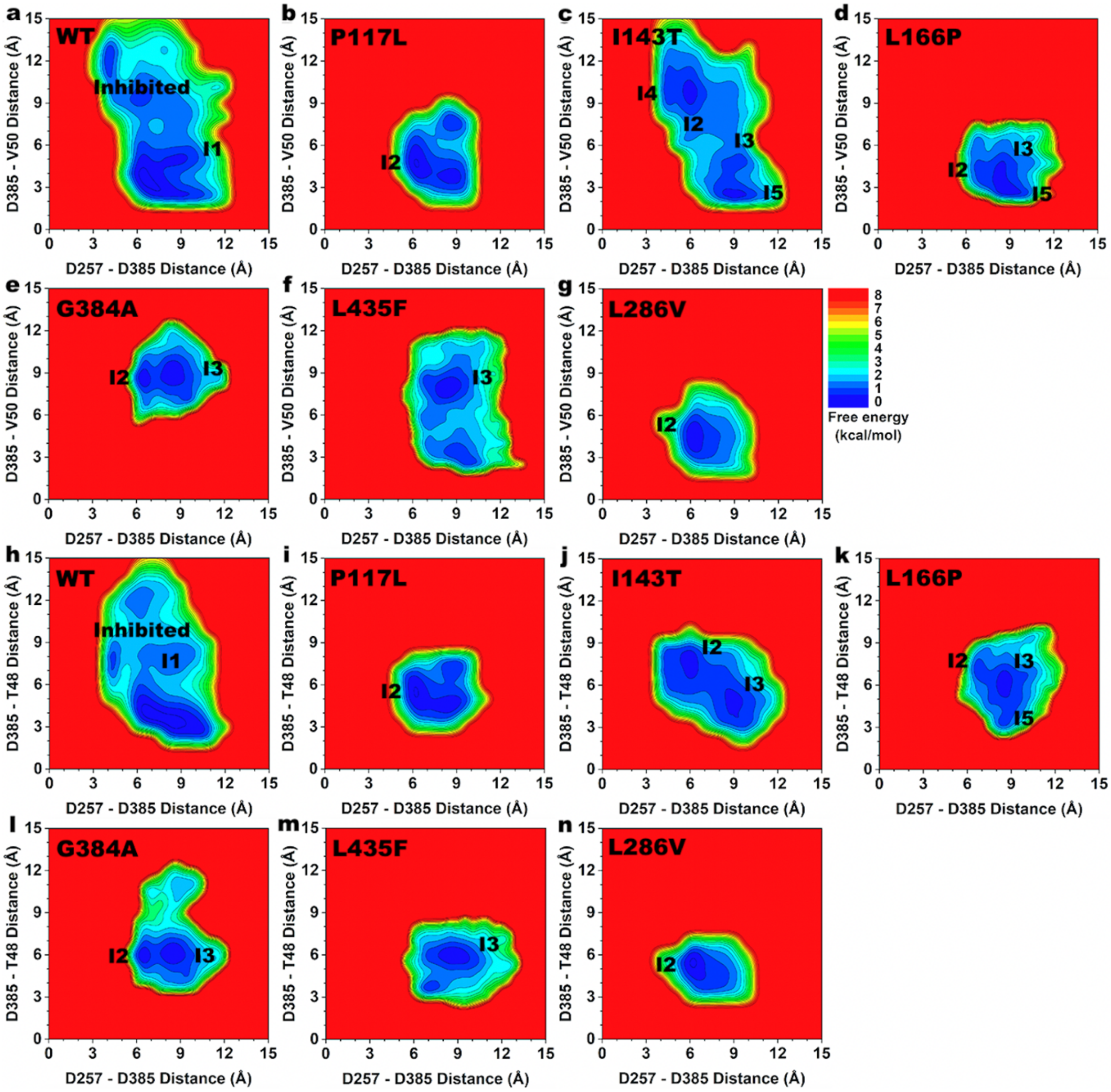

Supplementary Table 1. Summary of GaMD simulations performed on the APP-bound γ-secretase complexes, including WT, P117L, I143T, L166P, G384A, L435F, and L286V PS1 FAD mutant γ-secretase.

System	Method	Simulation Length (ns)	Boost Potential (kcal/mol)
**WT**	GaMD_Dual	3 x 1500	13.5 ± 4.3
**P117L**	GaMD_Dual	3 x 1500	11.3 ± 4.0
**I143T**	GaMD_Dual	3 x 1500	14.0 ± 4.4
**L166P**	GaMD_Dual	3 x 1500	14.8 ± 4.5
**G384A**	GaMD_Dual	3 x 1500	13.8 ± 4.4
**L435F**	GaMD_Dual	3 x 1500	14.0 ± 4.4
**L286V**	GaMD_Dual	3 x 1500	14.1 ± 4.4

Supplementary Table 2. pKa values of D257 and D385 in the starting structure (PDB: 6IYC) and low-energy conformational states from GaMD simulations as calculated using PROPKA3[1,2] at pH 7.0.

System	pKa(D257)	pKa(D385)
**Starting structure**	8.0	8.8
**"Active" (WT)**	5.6	9.8
**"Active" (L286V)**	6.0	10.1
**"Active" (P117L)**	6.3	9.0
**"Inhibited" (WT)**	4.7	10.1
**"I1" (WT)**	5.6	7.1
**"I2" (I143T)**	5.8	8.8
**"I3" (I143T)**	3.6	7.8
**"I4" (I143T)**	5.6	7.2
**"I5" (L166P)**	5.0	9.6

Supplementary Table 3. Summary of the (a) Cγ-atom distances between PS1 residues D257 and D385 and (b) Cγ-$C_β$-atom distances between PS1 residues D257 and A385 in the available PDB structures of γ-secretase on the Protein Data Bank.

PDB	Structure description on the Protein Data Bank	Year released	D257-D385 Distance (Å)
4UIS[(a)]	CryoEM structure of human gamma-secretase complex	2015[3]	4.0
5FN5[(a)]	CryoEM structure of gamma-secretase in class 3 of apo-state ensemble	2015[4]	5.4
5FN2[(a)]	CryoEM structure of gamma-secretase in complex with a drug DAPT	2015[4]	3.9
5FN4[(a)]	CryoEM structure of gamma-secretase in class 2 of the apo-state ensemble	2015[4]	11.5
5FN3[(a)]	CryoEM structure of gamma-secretase in class 1 of the apo-state ensemble	2015[4]	5.1
5A63[(a)]	CryoEM structure of human gamma-secretase complex at 3.4Å resolution	2015[5]	7.2
6IDF[(b)]	CryoEM structure of gamma-secretase in complex with a Notch fragment	2018[6]	8.6
6IYC[(b)]	Recognition of the amyloid precursor protein by human gamma-secretase	2019[7]	9.1
6LQG[(a)]	Human gamma-secretase in complex with small molecule Avagacestat	2021[8]	7.1
6LR4[(a)]	Molecular basis for inhibition of human gamma-secretase by small molecule	2021[8]	6.2
7C9I[(a)]	Human gamma-secretase in complex with small molecule L-685,458	2021[8]	6.6
7D8X[(a)]	CryoEM structure of human gamma-secretase in complex with E2012 and L685458	2021[8]	6.4

Supplementary Table 4. List of amino acid residues constituting the S1', S2', and S3' subpockets in the WT "Active", L286V "Active", P117L "Active", and L166P "I5" low-energy conformations of γ-secretase. The residues that are within 5 Å of APP substrate residues P1', P2', and P3' are listed in the table.

System	S1'		S2'		S3'	
**"Active"**	I287		L85^{TM1}	Y389^{TM7}	T281	
**(WT)**	K380		V379	T421^{TM8}	I287	
	L381		K380	L422^{TM8}	G378	
	G382		L381	L425^{TM8}	V379	
	D385^{TM7}		G382	L432	K380	
			D385^{TM7}	A434	L381	
**"Active"**	D257^{TM6}	G382	L85^{TM1}	T421^{TM8}	T281	
**(L286V)**	L268^{TM6a}	D385^{TM7}	V379	L422^{TM8}	L282	
	L271^{TM6a}	A431	K380	L425^{TM8}	I287	
	V272^{TM6a}	A434	L381	A431	V379	
	L282		G382	L432	K380	
	I287		D385^{TM7}	P433	L381	
	K380		Y389^{TM7}	A434	L425^{TM8}	
	L381		L418^{TM8}	L435	A431	
**"Active"**	D257^{TM6}	G382	R269^{TM6a}	L418^{TM8}	R269^{TM6a}	K380
**(P117L)**	L268^{TM6a}	D385^{TM7}	V272^{TM6a}	T421^{TM8}	L271^{TM6a}	L381
	R269^{TM6a}		V379	L422^{TM8}	V272^{TM6a}	G382
	L271^{TM6a}		K380	L425^{TM8}	A275^{TM6a}	
	V272^{TM6a}		L381	A434	Q276^{TM6a}	
	I287		G382	L435	I287	
	K380		D385^{TM7}	S438^{TM9}	G378	
	L381		Y389^{TM7}		V379	
**"I5"**	D257^{TM6}		L85^{TM1}	Y389^{TM7}	I287	
**(L166P)**	I287		V379	L418^{TM8}	G378	
	K380		K380	L422^{TM8}	V379	
	L381		L381	A434	K380	
	G382		G382	L435	L381	
	D385^{TM7}		D385^{TM7}	S438^{TM9}	G382	

Supplementary References

1 Sondergaard, C. R., Olsson, M. H. M., Rostkowsi, M. & Jensen, J. H. Improved Treatment of Ligands and Coupling Effects in Empirical Calculations and Rationalization of pKa values. *Journal of Chemical Theory and Computation* **7**, 2284-2295 (2011).

2 Olsson, M. H. M., Sondergaard, C. R., Rostkowsi, M. & Jensen, J. H. PROPKA3: consistent treatment of internal and surface residues in empirical pKa predictions. *Journal of Chemical Theory and Computation* **7**, 525-537 (2011).

3 Sun, L. *et al.* Structural Basis of Human Gamma-Secretase Assembly. *Proc Natl Acad Sci USA* **112**, 6003 (2015).

4 Bai, X. C., Rajendra, E., Yang, G., Shi, Y. & Scheres, S. H. Sampling the conformational space of the catalytic subunit of human gamma-secretase. *eLife*, 11182 (2015).

5 Bai, X. C. *et al.* An Atomic Structure of Human Gamma-Secretase. *Nature* **525**, 212 (2015).

6 Yang, G. *et al.* Structural basis of Notch recognition by human gamma-secretase. *Nature* **565**, 192-197 (2019).

7 Zhou, R. *et al.* Recognition of the amyloid precursor protein by human gamma-secretase. *Science* **363**, aaw0930 (2019).

8 Yang, G. *et al.* Structural basis of gamma-secretase inhibition and modulation by small molecule drugs. *Cell* **184**, 521-533.e514 (2021).
