# Supplementary material for: Effects of presenilin-1 familial Alzheimer’s disease mutations on γ-secretase activation for cleavage of amyloid precursor protein
Source: Commun Biol. 2023 Feb 14;6:174. doi: 10.1038/s42003-023-04539-1 (PMC9929099; doi:10.1038/s42003-023-04539-1)
Supplement: Supplementary file 3 — Description of Additional Supplementary Files [file 42003_2023_4539_MOESM3_ESM.pdf]

Description of Additional Supplementary Files

File name: Supplementary Data 1
Description: Representative PDB structures of low-energy conformational states obtained from GaMD simulations of APP-bound γ-secretase.
