# Supplementary material for: Effects of presenilin-1 familial Alzheimer’s disease mutations on γ-secretase activation for cleavage of amyloid precursor protein
Source: Commun Biol. 2023 Feb 14;6:174. doi: 10.1038/s42003-023-04539-1 (PMC9929099; doi:10.1038/s42003-023-04539-1)
Supplement: Supplementary file 5 — Reporting Summary [file 42003_2023_4539_MOESM5_ESM.pdf]

nature portfolio

Corresponding author(s): Michael S. Wolfe and Yinglong Miao

Last updated by author(s): Sep 6, 2022

Reporting Summary

Nature Portfolio wishes to improve the reproducibility of the work that we publish. This form provides structure for consistency and transparency in reporting. For further information on Nature Portfolio policies, see our Editorial Policies and the Editorial Policy Checklist.

Statistics

For all statistical analyses, confirm that the following items are present in the figure legend, table legend, main text, or Methods section.

n/a	Confirmed	
☐	☒	The exact sample size (*n*) for each experimental group/condition, given as a discrete number and unit of measurement
☐	☒	A statement on whether measurements were taken from distinct samples or whether the same sample was measured repeatedly
☒	☐	The statistical test(s) used AND whether they are one- or two-sided<br>*Only common tests should be described solely by name; describe more complex techniques in the Methods section.*
☐	☒	A description of all covariates tested
☒	☐	A description of any assumptions or corrections, such as tests of normality and adjustment for multiple comparisons
☐	☒	A full description of the statistical parameters including central tendency (e.g. means) or other basic estimates (e.g. regression coefficient) AND variation (e.g. standard deviation) or associated estimates of uncertainty (e.g. confidence intervals)
☒	☐	For null hypothesis testing, the test statistic (e.g. *F*, *t*, *r*) with confidence intervals, effect sizes, degrees of freedom and *P* value noted<br>*Give P values as exact values whenever suitable.*
☒	☐	For Bayesian analysis, information on the choice of priors and Markov chain Monte Carlo settings
☒	☐	For hierarchical and complex designs, identification of the appropriate level for tests and full reporting of outcomes
☐	☒	Estimates of effect sizes (e.g. Cohen's *d*, Pearson's *r*), indicating how they were calculated

Our web collection on statistics for biologists contains articles on many of the points above.

Software and code

Policy information about availability of computer code

Data collection	AMBER 2020 (https://ambermd.org), PROPKA3 (https://github.com/jensengroup/propka)
Data analysis	AmberTools (https://ambermd.org), PyReweighting (http://miaolab.org/PyReweighting/), PyMOL (https://pymol.org/), GNU Plot (http://www.gnuplot.info), Origin Pro (https://www.originlab.com/origin), GraphPad Prism (https://www.graphpad.com)

For manuscripts utilizing custom algorithms or software that are central to the research but not yet described in published literature, software must be made available to editors and reviewers. We strongly encourage code deposition in a community repository (e.g. GitHub). See the Nature Portfolio guidelines for submitting code & software for further information.

Data

Policy information about availability of data

All manuscripts must include a data availability statement. This statement should provide the following information, where applicable:
- Accession codes, unique identifiers, or web links for publicly available datasets
- A description of any restrictions on data availability
- For clinical datasets or third party data, please ensure that the statement adheres to our policy

All data generated or analysed during this study are included in this published article (and its supplementary information files), except that the all-atom simulation data generated during and/or analysed during the current study are available upon request from the corresponding author Dr. Miao.

Human research participants

Policy information about studies involving human research participants and Sex and Gender in Research.

Reporting on sex and gender	N/A
Population characteristics	N/A
Recruitment	N/A
Ethics oversight	N/A

Note that full information on the approval of the study protocol must also be provided in the manuscript.

Field-specific reporting

Please select the one below that is the best fit for your research. If you are not sure, read the appropriate sections before making your selection.

☒ Life sciences ☐ Behavioural & social sciences ☐ Ecological, evolutionary & environmental sciences

For a reference copy of the document with all sections, see nature.com/documents/nr-reporting-summary-flat.pdf

Life sciences study design

All studies must disclose on these points even when the disclosure is negative.

Sample size	All biological assays and molecular dynamics simulations are performed in triplicates.
Data exclusions	No data was excluded for the data analysis
Replication	All biological assays and molecular dynamics simulations are performed in triplicates.
Randomization	Three independent all-atom production simulations with randomized initial atomic velocities were performed on each system.
Blinding	N/A

Reporting for specific materials, systems and methods

We require information from authors about some types of materials, experimental systems and methods used in many studies. Here, indicate whether each material, system or method listed is relevant to your study. If you are not sure if a list item applies to your research, read the appropriate section before selecting a response.

Materials & experimental systems

n/a	Involved in the study	
☐	☒	Antibodies
☐	☒	Eukaryotic cell lines
☒	☐	Palaeontology and archaeology
☒	☐	Animals and other organisms
☒	☐	Clinical data
☒	☐	Dual use research of concern

Methods

n/a	Involved in the study	
☒	☐	ChIP-seq
☒	☐	Flow cytometry
☒	☐	MRI-based neuroimaging

Antibodies

Antibodies used	Monoclonal Anti FLAG M2 antibody produced in mouse, Milli-pore Sigma, Cat no. F3165
Validation	Monoclonal ANTI-FLAG® M2 antibody produced in mouse has been used in:<br>immunoblotting

Eukaryotic cell lines

Policy information about cell lines and Sex and Gender in Research

Cell line source(s)	Freestyle HEK 293 F cells commercially available from thermo fisher scientific cat. no R79007
Authentication	cell lines are not authenticated by us since these are commercially available
Mycoplasma contamination	No mycoplasma contamination test was performed by us since these are commercially available
Commonly misidentified lines (See ICLAC register)	*Name any commonly misidentified cell lines used in the study and provide a rationale for their use.*
